# Mid-term results of mitral valve palsty in patients with mitral sclerotic lesion

**DOI:** 10.1186/s13019-016-0473-4

**Published:** 2016-05-10

**Authors:** Masanori Hirota, Tadashi Isomura, Chieko Katsumata, Fusahiko Ito, Masazumi Watanabe

**Affiliations:** Department of Cardiovascular Surgery, Tokyo Metropolitan Hiroo Hospital, 10-34-2 Ebisu, Shibuya-ward, Tokyo, 150-0013 Japan

**Keywords:** Rheumatic valvular disease, Mitral valve plasty, Mitral stenosis

## Abstract

**Background:**

Mitral valve repair is preferred over prosthetic replacement. We surgically repaired mitral valve with degenerated sclerotic lesion and demonstrated mid-term results.

**Methods:**

Mitral valve plasty (MVP) was performed with several procedures including ring annuloplasty, leaflet slicing and decalcification.

**Results:**

There were 19 males and 19 females with a mean age of 67 ± 12 y.o (*n* = 38). All patients were successfully treated MVP except one case with unrepairable injuries of the thin leaflet. In patients underwent MVP (*n* = 37), MVP included ring annuloplasty with a rigid full ring of 32 ± 2 mm (*n* = 37), leaflet slicing (*n* = 37), decalcification (*n* = 15) and artificial chordae (*n* = 14). Mitral valve area was statistically enlarged by MVP (1.65 ± 0.57 vs 2.51 ± 0.58 cm^2^, *p* < 0.001). Left atrial diameter was statistically reduced after the operation (55 ± 10 vs 46 ± 9 mm, *p* < 0.001). Severity of MR and right ventricular systolic pressure (RVSP) were statistically decreased after the operation (MR; 1.8 ± 1.0 vs 0.7 ± 0.9, *p* < 0.001, RVSP; 38 ± 15 vs 30 ± 9 mm Hg, *p* < 0.001). There were 4 cases with residual MR (Grade II, *n* = 3; Grade III, *n* = 1). The 30-days mortality was 0 %. There was one late death due to non-cardiogenic cause (the 3-year survival rate of 97 %) and no redo case due to deterioration of the mitral valve during follow-up period of 21 ± 13 months.

**Conclusions:**

Successful mid-term survival and freedom from reoperation might expect to the durability of MVP in patients with mitral sclerotic lesion.

## Background

Rheumatic heart disease is one of degenerative valvular disorders, which mainly affect mitral valve in the young population [1]. Although mitral regurgitation (MR) was the most common lesion in the first and second decades, the relative prevalence of mitral stenosis (MS) increased with age [2]. The characteristic alteration is thickening of the leaflet secondary to inflammation, which results in sclerotic lesion including atherosclerotic calcification in the advanced cases [3]. Although the clinical presentation depends on the degenerative lesions such as MR, and MS and a mixed form, the definitive treatment is surgical correction despite age. According to earlier surgical reports, the superiority of mitral valve plasty (MVP) over mitral valve replacement (MVR) had been already established in patients with MR [4, 5]. However, MVP for sclerotic lesion is still challenging and the durability of the procedure had not been established.

Following earlier reports [6, 7], we have surgically treated patients with mitral sclerotic lesion. We retrospectively evaluated early and mid-term outcomes of MVP, and discussed details of technical pitfalls for such patients.

## Methods

### Patients

Between July 2011 and December 2014, patients with mitral sclerotic lesion treated by MVP were involved in this series. This study was approved by the Institutional Review Board of Tokyo Metropolitan Hiroo Hospital and informed consent was preoperatively obtained from all patients.

### Surgical indication

MVP was the first choice of procedures for mitral sclerotic lesion. However, we abandoned MVP for limited cases such as severe diffuse calcification of both mitral leaflets and mitral annular calcification (MAC). For limited cases requiring longer ACC time for MVP (>60 min), MVP was abandoned and converted to MVR during operation.

### Assessment of valvular morphology and functional parameters

Trans-thoracic echocardiography was used to evaluate functional and geometric parameters, including mitral valvular area (MVA), LV end-diastolic and -systolic diameter (LVEDD and LVESD), left atrial diameter (LAD), ejection fraction (EF), right ventricular systolic pressure (RVSP), and severity of MR and tricuspid regurgitation (TR).

### Surgical procedures

Briefly, after cardioplegic cardiac arrest, the LA was opened via right-sided left atriotomy. MVP was performed via the right-sided left atriotomy. MVP included combined mitral procedures such as ring annuloplasty, leaflet slicing, decalcification, artificial chordae and chordal cutting, following earlier reports [6, 7].

White excess membrane on the mitral leaflet was sharply sliced and bluntly dissected from the clear zone extending to the rough zone. It was removed to obtain flexibility of the sclerotic leaflets. Especially for advanced cases with atherosclerotic calcification, it was crushed by the Pean Forceps into small pieces, which were manually removed. After removal of calcification on the leaflet, thin fragile leaflet appeared beneath calcification. When the injuries of the mitral leaflet occurred, the tears were directly closed with polypropylene sutures. For cases with chordal shortening, it was cut to adjust the height of coaptation by artificial chordae with expanded polytetrafluoroethylene sutures (GORE-TEX CV-5; W. L. Gore & Associates, Inc, Flagstaff, Ariz). When the seagull sign was detected by preoperative echocardiography due to chordal shortening, the second chordae of both leaflets was cut to facilitate valvular mobility. The mitral orifice was enlarged with these techniques to pass the Hegar (25 mm in diameter). After mitral valvular procedures, ring annuloplasty was performed with a rigid full ring. For patients with Af, MAZE operation and closure of the LA appendage were concomitantly performed.

### Statistical analysis

Results are expressed as means ± SEM. An analysis was performed by paired Student’s *t* test to compare the data. The Kaplan-Meier survival method was used to calculate estimates for mid-term survival. The criterion for statistical significance was set at a value of *p* < 0.05.

## Results

There were 19 males and 19 females in this study (*n* = 38). The mean age was 67 ± 12 y.o, ranging from 25 to 89 y.o. All patients were Japanese (>50 y.o.) except two foreign patients (Nepalese 25 y.o., Iranian 49 y.o.). There were 31 patients with chronic or paroxymal atrial fibrillation (Af). There were 5 redo cases after mitral procedure, including open mitral commissurotomy (OMC; *n* = 2), closed mitral commissurotomy (CMC; *n* = 1), and percutaneous transvenous mitral commissurotomy (PTMC; *n* = 2).

All patients were successfully treated by MVP except one case with unrepairable injuries of the thin leaflet during operation. MVP was performed with various procedures including ring annuloplasty (*n* = 37), leaflet slicing (*n* = 37), decalcification (*n* = 15), artificial chordae (*n* = 14) and chordal cutting (*n* = 8). The ring size was 32 ± 2 mm, ranging from 28 to 34 mm. Concomitant procedures other than MVP included aortic valve replacement/plasty (AVR/AVP; *n* = 10/2), tricuspid annuloplasty (TAP; *n* = 26), MAZE operation (*n* = 24) and coronary artery bypass grafting (CABG; *n* = 3). In a total of 31 patients with Af, MAZE procedure was underwent in 24 cases and regular sinus rhythm was obtained in 16 cases (67 %). Aortic cross-clamping (ACC) and cardiopulmonary bypass (CPB) time were 131 ± 28 and 152 ± 31 min, respectively.

Echocardiographic parameters were summarized in Table 1. MVA was statistically enlarged by combined mitral valve procedures (1.65 ± 0.57 vs 2.51 ± 0.58 cm^2^, *p* < 0.001) and LAD was statistically reduced after the operation (55 ± 10 vs 46 ± 9 mm, *p* < 0.001). Severity of MR and RVSP were statistically decreased after the operation (MR; 1.8 ± 1.0 vs 0.7 ± 0.9, *p* < 0.001, RVSP; 38 ± 15 vs 30 ± 9 mm Hg, *p* < 0.001). However, there were 4 cases with residual MR (Grade II, *n* = 3; Grade III, *n* = 1) in this series.

**Table 1:** Echocardiographic parameters

	Before operation	After operation	*P* value
Left ventricular end-diastolic diameter (mm)	51 ± 6	46 ± 7*	*p* < 0.001
Left ventricular end-systolic diameter (mm)	33 ± 7	30 ± 6*	*p* < 0.001
Left atrial diameter (mm)	55 ± 10	46 ± 9*	*p* < 0.001
Mitral valvular area (cm2)	1.65 ± 0.57	2.51 ± 0.58*	*p* < 0.001
Ejection Fraction (%)	65 ± 8	63 ± 10	*p* = 0.118
Right ventricular systolic pressure (mm Hg)	38 ± 15	30 ± 9*	*p* < 0.001
Severity of mitral regurgitation	1.8 ± 1.0	0.6 ± 0.7*	*p* < 0.001

Values are expressed as means ± SEM

^*^ *P* < 0.05 vs. Before operation

**Fig. 1:**
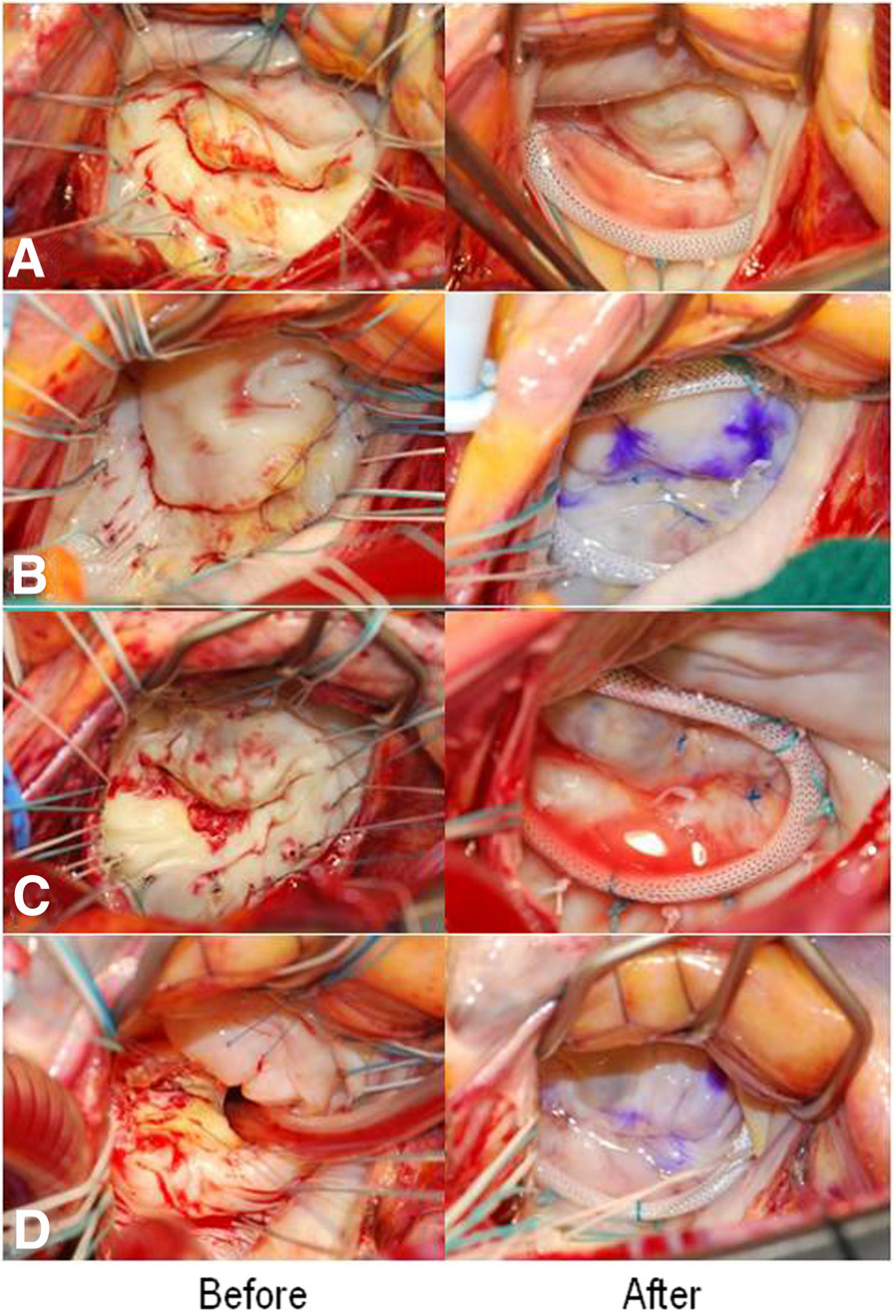
Representative appearances of the mitral leaflets before and after mitral valve plasty. White thick intima was covered with both mitral leaflets and there was localized calcification in the middle of the both leaflets and around the postero-median commissure (*left*). The thick leaflets became thin and the mobility of the leaflets increased after leaflet slicing (*right*) **a**. Both mitral leaflets included mild atherosclerotic lesion without commissural fusion **b**. The antero-laretal commissure was affected by advanced atherosclerotic calcification, which extended to the center of both leaflet **c**. In the most advanced case, the mitral leaflet around the antero-lateral commissure seemed to be destroyed by the broad severe atherosclerosis (*left*). However, thin mobile mitral leaflets appeared after careful decalcification and leaflet slicing (*right*) **d**

Macroscopic appearances of the mitral leaflets were shown in Fig. 1. Both mitral leaflets were thick in all cases (*n* = 38), and atherosclerotic calcification was detected in advanced cases (*n* = 15). Sclerotic lesion was surgically repaired with various procedures described above and deeper coaptation zone with thin mobile leaflets was created to obtain enlargement of MVA and to minimize residual MR.

The 30-days mortality was 0 %. However, there was one late death caused by cholecystitis during follow-up periods during a mean period of 21 ± 13 months. There was no case requiring mitral valve surgery due to deterioration of the mitral valve. As mid-term surgical results, the 3-year survival rate was 97 %. During follow-up, there was one patient with a history of re-admission due to congestive heart failure (CHF) caused by residual MR (Grade III) after MVP (2.7 %).

## Disscussion

We have surgically treated patients with mitral sclerotic lesion using combined various procedures, including ring annuloplasty, leaflet slicing, decalcification, artificial chordae and chordal cutting. To obtain larger mitral orifice, improvement of valvular mobility by slicing and decalcification is the most important issue for such patients. As a result, MVA was successfully enlarged by MVP and, early and mid-term surgical results were satisfactory. Accordingly, MAP would be one of the surgical options for patients with mitral sclerotic lesion.

Surgical repair for rheumatic valvular lesion had been started in the young population more than 20 years ago and the procedure is still a challenge for cardiac surgeons [8, 9]. In the early period of MVP for rheumatic valvular disease, the primary objective was avoidance of prosthetic implantation due to unavailability of prosthesis for infants and children. Recently, age of patients underwent MVP was gradually getting older and surgical candidates for MVP were extending to adult patients, who avoid warfarization due to expectancy for pregnancy or limitation of utilization of prosthesis in the developing and advanced countries [6, 10]. In regard to surgical result, MVP for rheumatic mitral valve had a superior long-term outcome compared with MVR in patients without commissural fusion [11]. Then, we challenged MVP in rheumatic mitral pathology and successfully repaired advanced mitral lesion in Japanese elder patients. Although the longest follow-up period was about 44 months after the operation at this time, mid-term results were acceptable in regard to survival and freedom from reoperation.

In patients with rheumatic mitral disease, various procedures such as leaflet slicing and decalcification were required for successful MVP because the mitral sclerotic lesion involved various and complex pathologies in both leaflets. Therefore, we utilized several procedures depending on the lesion to obtain larger MVA. However, attentions should be paid to prevent one of potential complications, i.e. residual MR, especially for patients with severe calcification at the commissure. Because residual MR would be one of the risk factors for intra-operative conversion to MVR and CHF requiring re-admission rather than MS. In our series, there were two cases requiring additional treatments due to residual MR. One case necessitated conversion to MVR due to unrepairable injuries of the thin leaflet after slicing during operation. Another case with CHF re-admitted for medical treatment due to postoperative MR around the commissure, in which aggressive removal of severe calcification had been performed. Careful handling of the thin leaflet after leaflet slicing would be one of the most important issues to improve surgical outcome. Additionally, commissurotomy for calcified commissure was not necessary, because effective MVA was obtained by natural coaptation by native leaflets after careful decalcification and leaflet slicing. Although the leaflet extension is also one of the effective procedures for rheumatic degenerative lesion, we had not applied for such fragile leaflet after leaflet slicing to avoid unintended injuries [12]. By our surgical strategy, mitral sclerotic lesion would be repairable even if the extent of calcification was severe, but localized.

Although we suggested the feasibility of MVP in patients with sclerotic lesion, there is no golden standard to select potential candidates for MVP [13]. The major reason to abandon MVP would be the extent of calcification, requiring higher technical difficulties. In case with several concomitant procedures, operative time for MVP should be limited to prevent longer ACC time. For these reasons, severe diffuse (not localized) calcification on both mitral leaflets was our relative contraindication. Redo cases following OMC, CMC and PTMC were not our absolute contraindication and another report also supported this idea [14]. Actually, we selected patients by our impression of preoperative echocardigraphic images and intraoperative findings of the mitral valve. As a result, MVA of 1.65 ± 0.57 cm^2^ was surgically repaired in this series. However, in patient without our indication of MVP (candidates for MVR), MVA of 1.14 ± 0.33 cm^2^ was significantly lower compared with this series. Although larger preoperative MVA would be related to successful outcome for MVP, the most important issue for selection of procedure would be the distribution of calcification, i.e., eccentricity of calcification. Thus, MVP would be feasible for patients with severe localized calcification of both mitral leaflets unless diffuse calcification of both mitral leaflets creates circular and fixed calcified orifice such as fish mouth.

There are several limitations to this study. Firstly, durability of our procedure had not been established because follow-up period was not long enough. Additionally, our results were obtained from retrospective analysis of only 38 patients. Further cases and follow-up period were required to validate data. Secondly, selection of procedure (MVP vs MVR) depended on our impression by preoperative echocardiographic images and intraoperative findings, which may include selection bias of procedure and affect the data. Thirdly, the operative patients in our series were much elder compared with other reports. For such complicated valvular lesion, MVR might include beneficial effects on early and long-term survival due to shorter ACC and CPB time. A randomized controlled trial is necessary to conclude the superiority of MVP for such patients.

## Conclusion

We surgically repaired rheumatic mitral valve with advanced degenerative sclerotic lesion with various procedures. Successful mid-term survival and freedom from reoperation might expect the durability of MVP.

## Competing interest

The authors declared that they have no competing interests.

## Authors’ contribution

MH carried out preparing manuscript. Dr. TI performed statistical analysis. Dr. CK, FI and MW collected the data. All authors read and approved the final manuscript.
